# Total recovery of visual acuity in a pediatric patient with compressive optic neuropathy secondary to sphenoid sinus mucocele

**DOI:** 10.5935/0004-2749.2021-0312

**Published:** 2022-10-19

**Authors:** Facundo Urbinati, Rahul Rachwani-Anil, Francisco Zamorano Martín, Guillermo Luque Aranda, Julia Escudero Gómez

**Affiliations:** 1 Departamento de Oftalmología Infantil, Hospital Regional de Málaga, Hospital Materno Infantil, Málaga, Spain; 2 Hospital Regional de Málaga, Hospital Civil, Málaga, Spain; 3 Departamento de Oftalmología, Hospital de Antequera, Málaga, Spain

**Keywords:** Sphenoid sinus, Mucocele, Orbital diseases, Optic nerve diseases, Nervous system diseases, Neuroimaging, Visual acuity, Child, Seio esfenoidal, Mucocele, Doenças orbitárias, Doenças do nervo óptico, Doenças do sistema nervoso, Neuroimagem, Acuidade visual, Criança

## Abstract

We present an unusual case of a 13-year-old male pediatric patient with a diagnosis of sphenoid sinus mucocele. The patient suffered a progressive loss of visual acuity over three months followed by a total recovery of his visual acuity after surgery. The patient presented at the emergency room complaining of progressive loss of visual acuity in his left eye which decreased to hand motion over the preceding months. Imaging studies revealed a cystic mass, suggestive of sphenoid sinus mucocele, which was causing compressive optic neuropathy and proptosis. The patient was scheduled for a sphenoidectomy and resection of the mass. Three days after surgery, the patient’s visual acuity in the left eye was 20/20, indicating complete recovery from his symptoms. We suggest that the excellent outcome in this patient may be attributable to his age. His ongoing physical development might have been the decisive factor in the recovery of his visual acuity following compressive optic neuropathy secondary to sphenoid sinus mucocele. Further research is needed to verify this proposed explanation.

## Abstract (pt)

Apresentamos um caso incomum de paciente pediátrico com diagnóstico de mucocele de seio esfenoidal, que apresentou perda progressiva da acuidade visual ao longo de três meses, resultando em recuperação total da acuidade visual após a cirurgia. Paciente do sexo masculino, 13 anos, procurou o pronto-socorro, queixando-se de perda progressiva da acuidade visual do olho esquerdo nos últimos três meses. Exames de imagem revelaram uma massa cística sugestiva de mucocele de seio esfenoidal, causando neuropatia óptica compressiva e proptose. O paciente foi agendado para esfenoidectomia e ressecção da massa. Três dias após a cirurgia, a acuidade visual do paciente no olho esquerdo era de 20/20, apresentando recuperação completa dos sintomas. Diante dos resultados de nosso paciente, sugerimos que a idade do paciente pode ser decisiva na recuperação da acuidade visual de uma neuropatia óptica compressiva secundária à mucocele de seio esfenoidal. Mais pesquisas são necessárias para verificação desses dados.

## INTRODUCTION

Mucoceles are benign local encapsulated masses found in the paranasal sinuses. They contain mucous and are covered by epithelium. They are believed to be a consequence of sinus obstruction. The sphenoid sinuses are the rarest location for these masses, representing only 1% of all sinus mucoceles^(1)^. The most common presenting symptom is headache, which manifests in 87% of cases, but ophthalmic manifestations are the second most prevalent, at 85%, particularly ophthalmoplegia. There may also be visual defects, such as visual acuity (VA) impairment, double vision, visual field defects, and proptosis^(2^,^3)^.

To the best of our knowledge, the available literature regarding sphenoid mucocele in pediatric patients consists of just a few case reports. We report a case of a pediatric patient who presented with a complaint of VA impairment and was subsequently diagnosed with sphenoid sinus mucocele (SSM) and secondary compressive optic neuropathy. VA was hand motion. After surgery, the patient’s VA quickly returned to 20/20, representing a complete recovery.

## CASE REPORT

We present the case of a 13-year-old male pediatric patient who presented to the emergency room complaining of left eye (LE) VA impairment that had developed over the past 3 months along with periorbital pain. The patient’s description indicated that his visual symptoms had begun as a central scotoma that had widened over the weeks.

Exploration revealed a discrete palpebral fissure asymmetry (right eye [RE]: 10 mm; LE: 11 mm). The patient presented with orthophoria in the primary position. His extraocular muscle movements in the nine gaze positions were normal, ruling out a diagnosis of paresis or restriction. He said that he had not experienced double vision. An ocular examination revealed a Marcus Gunn pupil in the LE and a poorly reactive pupil in the RE.

**Figure 1:**
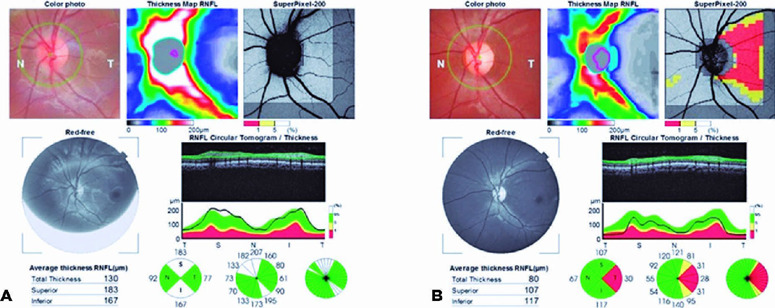
Preoperative and postoperative optic coherence tomography (OCT) of the left eye of a pediatric patient with compressive optic neuropathy secondary to sphenoid sinus mucocele. (A) Preoperative OCT of the left eye including a retinal nerve fiber layer (RNFL) map. Thickening of the superior and inferior sectors was observed. (B) Preoperative OCT of the left eye including an RNFL map. A reduction in the size of the superior and inferior sectors was seen, as well as a significant reduction in the temporal RNFL sectors.

A slit-lamp examination revealed the anterior segment of both eyes to be normal. Funduscopy of the RE was normal but the LE had an elevated optic disc in its inferior aspect. Optic coherence tomography (OCT) of the LE showed thickening of the superior and inferior retinal nerve fiber layers (RNFL). OCT of the RE was within normal limits (Figure 1A).

**Figure 2:**
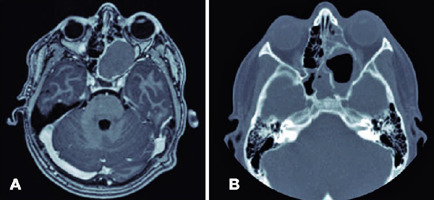
Preoperative magnetic resonance imaging (MRI) and postoperative computed tomography (CT) of the head of a pediatric patient with compressive optic neuropathy secondary to sphenoid sinus mucocele. (A) Preoperative head magnetic resonance imaging (MRI). A cystic mass was found in the left sphenoid sinus, sized 39.91 mm x 25.55 mm x 51 mm. The mass was causing left globe proptosis and deformation of the left orbital medial wall without bone erosion. It was also compressing the optic nerve. (B) Postoperative head computed tomography (CT). A hollow cavity can be seen at the surgical site with a hydro-air level in the anterior aspect and surgical debris in the posterior aspect. The deformation of the orbital medial wall remained but the globe was no longer compressed.

Subsequently, computed tomography (CT) and magnetic resonance imaging (MRI) scans of the patient’s head were performed, resulting in the discovery of a unique cystic mass of benign appearance with dimensions of 39.91 mm x 25.55 mm x 51 mm. The mass occupied the left sphenoid, left maxillary, and ipsilateral posterior ethmoidal sinuses, displacing and deforming the intraconal area of the left orbit. This suggested SSM (Figure 2A). The mass was also compressing the optic nerve and causing left globe proptosis, deforming the medial orbital wall but without bone erosion. The mass had not infiltrated the vascular, muscular, or nervous tissue. The optic nerve chiasma was unaffected.

The mass was well defined, with smooth thin walls and peripheral enhancement. No diffusion restrictions were found, ruling out a dermoid or epidermoid tumor. The CT and MRI echo gradient sequences indicated the absence of calcium and there was no internal bleeding or vascular involvement.

Surgical treatment was performed in the Department of Head and Neck Surgery of Regional University Hospital of Málaga. This consisted of a left sphenoidectomy. During the procedure, the anterior wall of the sinus was broadly resected to aspirate the mucous content within. The pathology report confirmed our diagnosis of SSM, allowing us to rule out malignancy.

Postoperative LE OCT revealed a reduction in the thickness of the superior and inferior RNFL, as well as a significant reduction in the RNFL of the temporal sector (Figure 1B).

A postoperative CT scan of the patient’s head revealed residual bone remodeling. The hollow cavity comprised two parts divided by high-density lobulated postsurgical debris and a hydro-air level in the external and anterior aspects. The distortion to the medial orbital wall had rectified slightly and the compression on the orbital cone was relieved. There were no intracranial or extracranial postoperative complications (Figure 2B).

Three days after surgery, the patient exhibited a best-corrected visual acuity (BCVA) of 20/20 in the LE, his Ishihara color test score was 14/15, and the pupillary reflex had returned to normal. Moreover, the patient was no longer experiencing any pain or other symptoms. Funduscopy revealed an improvement in the optic disc elevation. At the time of writing, it has been 12 months since the patient’s surgery. He currently maintains a BCVA of 20/20 and the results of all ophthalmological examinations are normal.

## DISCUSSION

The visual impairment seen in patients with SSM is caused by ischemia and inflammation of the optic nerve due to its displacement and compression^(4)^. The expansion of these masses can disturb, distort, or compress nearby tissue and even cause bone erosion^(5)^. If the mass causes arterial obstruction or venous congestion, there may be ischemia of the optic nerve^(6)^.

Previous research regarding the prognosis for vision recovery in SSM patients has failed to find correlations between prognosis and variables that seem sure to have some effect^(7)^. Li et al.^(3)^ affirmed that visual recovery is variable even with prompt diagnosis and early surgery. They observed several cases in which prompt surgical treatment (<24 hours) did not improve the visual prognosis of the patient. Among the 32 cases included in their review, only three patients completely recovered VA. These patients were aged 22–28 with preoperative VA <0.1. One of these cases underwent surgery in the first 72 hours after symptom onset. Methylprednisolone was given before and after surgery, and penicillin G, metronidazole, and chloramphenicol were administered. The other two cases underwent surgery on days 11 and 34, respectively, and only methylprednisolone was administered preoperatively. The rest of the 32 cases showed little or no VA improvement after surgery. The authors of the study concluded that there seems to be no concordance between final VA prognosis and early surgical treatment^(3)^.

Carlson et al.^(7)^ analyzed the factors predictive of VA recovery after optic nerve decompression in chronic compressive neuropathy and included mucocele cases in their meta-analysis. Surprisingly, age did not seem to affect post-surgical VA prognosis, although it is known that older adults have higher postoperative morbidity rates^(7)^. However, the size of the mass does seem to affect VA prognosis after surgery, especially as larger masses may result in a greater diversion of the blood supply to the nerve^(7)^. Time is also an important factor and it has been proposed that surgery outcomes may be worse if performed more than a year after diagnosis. There is also a linear relationship between preoperative VA and postsurgical VA outcomes ^(7)^. Our patient presented with several poor prognostic indicators, including low preoperative VA and large lesion size. Nevertheless, he fully and rapidly recovered VA. Therefore, we hypothesize that pediatric patients may experience an enhanced recovery after surgery.

Regarding the type of lesion, Sethi et al. have published a series of cases of isolated sphenoid pathologies, including sphenoid sinusitis, sphenoid mucoceles, inflammatory sphenochoanal polyp, inverting papilloma, invasive pituitary adenoma, carcinoma, aspergilloma, and fibrous dysplasia. They concluded that isolated sphenoid disease is underdiagnosed but can usually be managed endoscopically^(5)^.

To our knowledge, there are few reported cases of pediatric sphenoid mucoceles with cranial nerve involvement. Endoscopic resection in other cases has resulted in the restoration of nerve function^(8)^.The only exceptions seem to be attributable to delayed intervention^(8)^. Despite the lack of large studies of other compressive tumors in pediatric patients, it seems reasonable to assume that larger tumors would lead to more devastating prognoses.

The treatment of SSM is carried out by endoscopic endonasal resection. This approach is considered the gold standard due to better visualization of the sphenoid sinus along with better restoration of respiratory function^(9)^. This minimally invasive technique involves resecting the mucocele by disrupting its walls and aspirating its contents to prevent future relapse. The most frequent complication resulting from this procedure is the formation of fibrosis that compresses or obstructs the surrounding tissue^(10)^.

Our patient suffered from impaired VA of hand movements for 3 months. Postoperatively, his BCVA had improved to 20/20 72 hours after surgery. The incidence of SSM is low and there is currently scarce literature on this pathology in pediatric patients. However, we suggest that our patient’s youth may have contributed to his rapid recovery despite the large size of the SSM and the lateness of intervention. Further research is needed into the recovery of visual acuity recovery after compressive optic neuropathy secondary to SSM.
